# Supplementary material for: Precise capture and dynamic relocation of nanoparticulate biomolecules through dielectrophoretic enhancement by vertical nanogap architectures
Source: Nat Commun. 2020 Jun 4;11:2804. doi: 10.1038/s41467-020-16630-w (PMC7272609; doi:10.1038/s41467-020-16630-w)
Supplement: Supplementary file 1 — Supplementary Information [file 41467_2020_16630_MOESM1_ESM.docx]

Supplementary Information

**Precise capture and dynamic relocation of nanoparticulate biomolecules through dielectrophoretic enhancement by vertical nanogap architectures**

Yu et al.

**Supplementary Note 1: Model for numerical simulations.**

Throughout the simulation, the two dimensional (2D) cross-sectional geometry of a single unit cell of VNE (**Supplementary Fig. 1**) was adopted and calculated using COMSOL Multiphysics (COMSOL Inc.). The diameter of the hole pattern (*L*) and the periodicity (*P*) between each pattern were set as *L* = 10 μm and *P* = 30 μm. The vertical geometric parameters and other material properties, including film thickness, permittivity (*ε*), electrical conductivity (*σ*), and thermal conductivity (*k*), were utilized in the simulation (**Supplementary Table 1**).

| Material | Film thickness | Permittivity (*ε*) | Electrical conductivity (*σ*)  [S·m^-1^] | Thermal conductivity (*k*) [W·K^-1^·m^-1^] |
| --- | --- | --- | --- | --- |
| Water | *t*_water_ = 200 μm | 80·*ε*_0_ [[1](#_ENREF_1)] | 10^-4^ (measured) | 0.6 [[2](#_ENREF_2)] |
| Au | *t*_Au_ = 40 nm | - | 4.9 × 10^7^ [[3](#_ENREF_3)] | 318 [[3](#_ENREF_3)] |
| PVP | *d* = 100 nm | 4.7·*ε*_0_ [[4](#_ENREF_4)] | 10^-13^ [[5](#_ENREF_5)] | 0.2 [[6](#_ENREF_6)] |
| ITO | *t*_ITO_ = 40 nm | - | 10^6^ [[7](#_ENREF_7)] | 10 [[8](#_ENREF_8)] |
| Glass | *t*_sub_ = 1 mm  *t*_cover_ = 100 μm | 4.6·*ε*_0_ [[9](#_ENREF_9)] | 10^-11^ [[10](#_ENREF_10)] | 1 [[3](#_ENREF_3)] |

**Supplementary Table** **1.** **Material properties and layer thicknesses for numerical simulations**


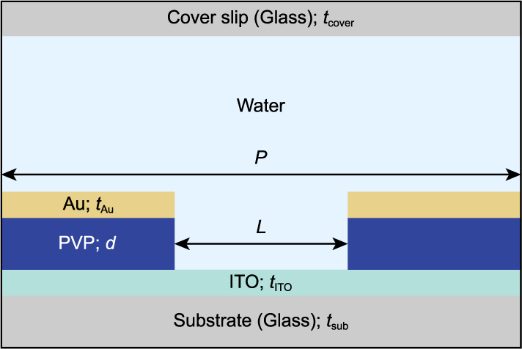


**Supplementary Figure** **1. Schematic of the simulation model.** Materials and geometric parameters of the numerical model of a single unit VNE for simulations.

**Supplementary Note 2: Simulations of DEP and ACEO.**

**Simulations of potential distribution on EDL.** Under an electrical potential, oppositely charged ions in an aqueous medium are attracted toward the electrodes and form an electric double layer (EDL), a thin layer of induced charges. In contrast to the well-established theory and related equations on horizontal nanogap electrodes (HNE)[^11^](#_ENREF_11), the behaviour of EDL on VNEs has not been reported yet. Herein, we set a simple simulation condition to estimate the EDL phenomenon on our VNEs (**Supplementary Fig. 2a**).With a voltage applied (amplitude of *V*_0_ and frequency of *f*), the equation of surface charge conservation at the interface between the EDL and the bulk water can be described as[^12^](#_ENREF_12)

 (1)

where *ϕ*, *ω*, and *C*_DL_ represent potential, angular frequency (*ω* = 2π*f*), and the surface capacitance per unit area of the EDL, respectively (i = $\sqrt{\text{-1}}$). From the Debye–Huckel theory[^13^](#_ENREF_13), it is estimated that *C*_DL_ = *ε*_m_/*λ*_D_, in which *λ*_D_ represents the Debye length (*λ*_D_ = 300 nm for low conductive medium). The potential at the EDL interface from equation (2) was applied to the electrode surface for ease of calculation; the potential boundary conditions of *ϕ* = *V*_0_ + *σZ*_DL_(*∂ϕ*/*∂y*) and *ϕ* = *V*_0_ + *σZ*_DL_(*∂ϕ*/*∂x*) were applied to the electrode surfaces parallel to the *x*- (red and blue lines) and *y*-axis (yellow lines), respectively, where *Z*_DL_ = 1/i*ωC*_DL_. As a boundary condition, root-mean-squared value of *V*_a_ (= + *V*_pp_/2$\sqrt{2}$; red and yellow lines) and ground condition of *V*_b_ (= 0; blue line) were applied to *ϕ*_a_ and *ϕ*_b_ of the top and bottom electrodes, respectively, to calculate the time-averaged results. While the upper boundary was specified as the Dirichlet condition (*ϕ* = 0; purple line) because of its significantly large separation from the electrodes[^14^](#_ENREF_14), the Neumann boundary condition (*∂ϕ*/*∂n* = 0; green lines) was applied to the left/right ends and boundaries along the nanogap. This is because the Neumann condition can be assigned to the symmetric boundaries and the interface between water and the dielectric[^15^](#_ENREF_15). Based on these boundary conditions, electric simulation was solved by applying Laplace’s equation (∇^2^*ϕ* = 0) to the bulk medium.

**Simulations of DEP.** Dielectrophoresis (DEP) is a phenomenon in which a force is exerted on a dielectric particle subjected to a non-uniform electric field (E-field; **E**). When an AC bias is applied with a particular frequency of *f* (*ω* = 2π*f*) and a peak-to-peak voltage amplitude of *V*_pp_, the time-averaged DEP force (**F**_DEP_) exerted on a suspended particle of radius *R* is given by[^16^](#_ENREF_16)

$F_{DEP}=\pi\varepsilon_{m}R^{3}\mathrm{Re}\left[f_{CM}(\omega)\right]\cdot\nabla E_{rms}^{2}$, (2)

where *ε*_m_ and Re[*f*_CM_(*ω*)] represent the permittivity of the medium and the real part of the Clausius–Mossotti (CM) factor, respectively. Note that the calculation of the CM factor will be described below (**Supplementary Note 10** in details). Since **F**_DEP_ is proportional to the root-mean-squared field gradient (∇**E**^2^_rms_), potential (*ϕ*) distribution should be calculated to analyse DEP behaviour. From the solution of *ϕ*, ∇**E**^2^ distribution was calculated using the E-field, derived from **E** = –∇*ϕ*. Thereby, **F**_DEP_ can be obtained from equation (2).

**Simulations of ACEO.** The interaction between the tangential components of the E-field and the induced charge of EDL generates fluidic motions, called AC electro-osmosis (ACEO). The ACEO fluidic flow of **u** = (*u*, *v*) with horizontal (*u*) and vertical velocity (*v*) was calculated using the fluid-mechanic numerical model (**Supplementary Fig. 2b**). Assuming that only the tangential component of slip velocity exists on the interface between the EDL and the bulk medium, the slip ACEO velocity condition at the interface can be given by[^17^](#_ENREF_17)^,^[^18^](#_ENREF_18)

 and *v* = 0 (3)

for the interface parallel to the *x*-axis (red and blue lines) and for the interface parallel to the *y*-axis (yellow lines),

*u* = 0 and (4)

where *η* and *Λ* represent the dynamic viscosity of water (8.9 × 10^-4^ Pa·s) and impedance ratio of the diffuse layer to that of the total double layer. The value of *Λ* was assumed to be 0.2 with the simplest numerical model with low conductivity[^18^](#_ENREF_18), and the slip velocity conditions of equations (3) and (4) were also applied to the electrode surfaces. In addition to the slip boundary conditions on the electrodes, the boundary conditions of no-slip (**u** = 0; purple lines) and symmetry (*u* = 0, *∂v*/*∂n* = 0; green lines) were assigned to the solid/water interfaces and left/right ends. Neglecting the inertial terms, the flow velocity of ACEO was evaluated by the bulk conditions of the incompressible Navier–Stokes equation[^19^](#_ENREF_19) along with the equation of mass conservation, ∇·**u** = 0,

$\eta\nabla^{2}u-\nabla p=0$ (5)

where *p* represent pressure.


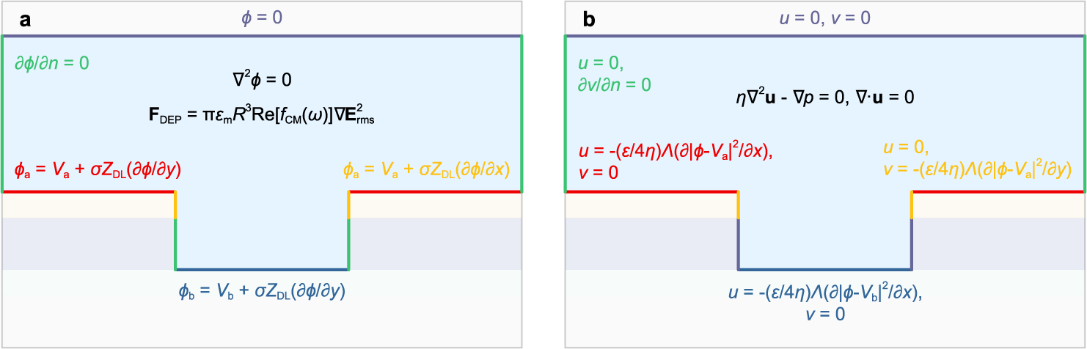


**Supplementary Figure** **2. Schematics of the simulation for potential on EDL with DEP and ACEO.** **a**, **b** Boundary and bulk conditions for simulation of potential on EDL with DEP (**a**) and ACEO (**b**).

**Supplementary Note 3: Simulations of electrothermal flow.**

**Simulations of Joule heating.** To investigate the Joule heating phenomenon in terms of absolute temperature (*T*), we adopted a simulation model that covers the entire structure (**Supplementary Fig. 3a**). Prior to the thermal simulation, the E-field distribution was calculated using an electrostatic numerical model. Although the electric simulation model for Joule heating is similar to that for DEP described above (**Supplementary Fig. 2a**), it utilizes the entire structure of the device with simple voltage boundary conditions (*ϕ*_a_ = *V*_a_ and *ϕ*_b_ = *V*_b_; red and yellow lines). Note that these conditions are also utilized to calculate ∇**E**^2^, which helps to evaluate the dielectric performance (**Main Figs. 1d-g**). For the thermal simulation, the Dirichlet boundary condition at room temperature (*T* = 298.15 K; purple lines) was assigned to the upper and lower boundaries, assuming that the outer region of the structure is maintained at room temperature. Further, the Neumann boundary condition (*∂T*/*∂n* = 0; green lines) was established for left–right symmetry. Finally, the temperature (*T*) distribution can be expressed as follows by applying the previously obtained electrical solution and simplified energy balance equation to the medium[^11^](#_ENREF_11):

$k\nabla^{2}T+\sigma E^{2}=0$ (6)

**Simulations of electrothermal flow.** Electrothermal flow (ETF) is a fluidic motion that arises from the electric forces induced by non-uniform thermal distribution. Therefore, electric, thermal, and subsequent fluidic simulations should be evaluated to analyse and estimate the electrothermal flow. Previous studies have demonstrated that temperature gradient (∇*T*) from localized heating leads to the gradient of permittivity (∇*ε*) and conductivity (∇*σ*) in the medium; ∇*ε* = (*∂ε*/*∂T*)·∇*T* and ∇*σ* = (*∂σ*/*∂T*)·∇*T*, respectively[^11^](#_ENREF_11). From these gradients, combined electric force (**F**_e_) of Coulomb and dielectric forces is generated, which can be expressed as[^20^](#_ENREF_20)

$F_{e}=\frac{1}{2}\cdot\frac{\varepsilon(\alpha-\beta)}{1+(\omega\varepsilon/\sigma)^{2}}\left(\nabla T\cdot E\right)E-\frac{1}{4}\varepsilon\alpha\left|E\right|^{2}\nabla T$ (7)

where *α* = (*∂ε*/*∂T*)/*ε* = -0.4 %·K^-1^ and *β* = (*∂σ*/*∂T*)/*σ* = 2.0 %·K^-1^, respectively[^21^](#_ENREF_21). Neglecting the inertial term, the flow velocity **u** of the horizontal velocity *u* and vertical *v* was then calculated using the incompressible Navier–Stokes equation with external forces (**F**_e_)[^19^](#_ENREF_19) and the equation of mass conservation, ∇·**u** = 0.

$\eta\nabla^{2}u-\nabla p+F_{e}=0$ (8)

Therefore, after obtaining **F**_e_ from the electric and thermal simulations, electrothermal flow velocity distribution can be evaluated by applying equation (8) to the bulk condition of the fluid mechanical simulation model. In this simulation model (**Supplementary Fig. 3b**), the boundary conditions of solids/water interfaces were designated as no-slip conditions (**u** = 0; red lines), whereas the symmetric boundary condition (*u* = 0, *∂v*/*∂n* = 0; green lines) were considered for the left and right ends.


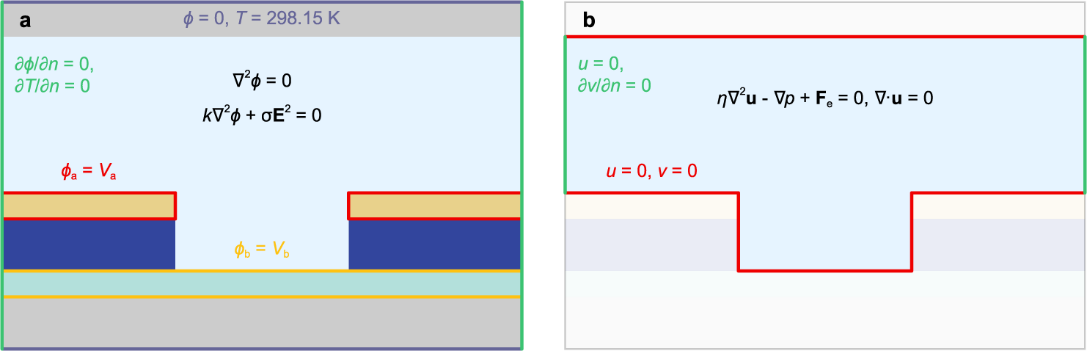


**Supplementary Figure** **3. Schematics of the simulation conditions for Joule heating and ETF.** **a**, **b** Boundary and bulk conditions for the simulation of Joule heating (**a**) and ETF (**b**).

**Supplementary Note** **4: Simulation results of F_DEP_, F_ACEO_, and F_ETF_ (u_DEP_, u_ACEO_, and u_ETF_).**

For the quantitative comparison of simulation results, the electrohydrodynamic drag forces acting on the particle owing to ACEO (**F**_ACEO_) and ETF (**F**_ETF_) are calculated using **F** = -6π*ηR*(**u**_p_ - **u**_m_), in which **u**_p_ and **u**_m_ represents particle velocity and fluid velocity induced by ACEO (**u**_ACEO_) and ETF (**u**_ETF_), respectively. For calculating **F**_ACEO_ and **F**_ETF_, **u**_p_ = 0 was initially assumed, indicating that electric potential has just been applied, as mentioned in the main manuscript[^22^](#_ENREF_22). Meanwhile, DEP-induced particle velocity (**u**_DEP_ = **F**_DEP_/6π*ηR*) is also calculated and compared with **u**_ACEO_ and **u**_ETF_. These results (**Supplementary Fig. 4**) show that ETF is negligible (in the order of 10^-18^ N and 10^-10^ m·s^-1^) compared with DEP and ACEO (in the order of 10^-18^ N and 10^-10^ m·s^-1^).


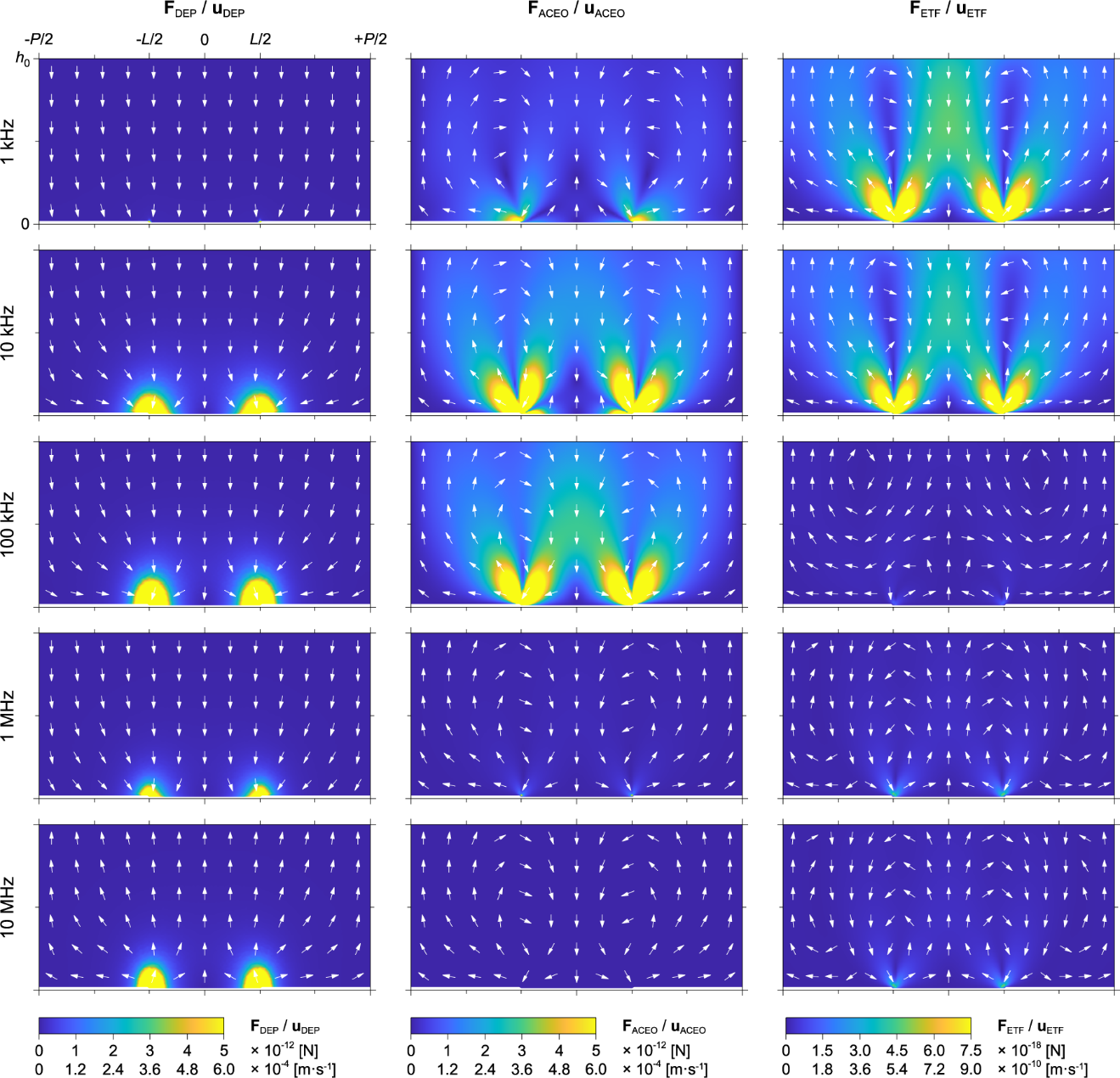


**Supplementary Figure** **4. F_DEP_, F_ACEO_, and F_ETF_ (u_DEP_, u_ACEO_, and u_ETF_) on VNE under various AC frequencies.** Cross-sectional simulations of **F**_DEP_ (and **u**_DEP_; left column), **F**_ACEO_ (and **u**_ACEO_; middle column), **F**_ETF_ (and **u**_ETF_; right column) of VNE (*d* = 100 nm) exerting on 1 μm PS particles under various frequencies; *V*_pp_ = 2.5 V, *f* = 1 kHz (first row), 10 kHz (second row), 100 kHz (third row), 1 MHz (fourth row), and 10 MHz (fifth row). Note that the colours and arrows indicate the magnitude and direction of forces and velocities (*h*_0_ = 10 μm).

**Supplementary Note 5: Calculation of gravitation, buoyancy, and Brownian motion.**

**Calculation of gravitation and buoyancy.** The gravitational force (**F**_grav_) and buoyant force (**F**_buoy_) acting on a suspended particle of radius *R* can be expressed as

$F_{grav}+F_{buoy}=\frac{4}{3}\pi R^{3}(\rho_{p}-\rho_{m})g$ (9)

where **g**, *ρ*_p_, and *ρ*_m_ denote the gravitational acceleration (9.81 m·s^-2^) as well as density and medium of the particle, respectively. Under an aqueous environment (*ρ*_m_ = 0.997 g·ml^-1^), the force exerted on the PS particles (*ρ*_p_ = 1.05 g·ml^-1^) was calculated to be 2.7 × 10^-16^ N, 2.2 × 10^-18^ N, 2.7 × 10^-19^ N, and 3.4 × 10^-20^ N for PS particles of 1 μm, 200 nm, 100 nm, and 50 nm, respectively. As a result, gravitation and buoyancy were found to be negligible compared with DEP and ACEO (**Supplementary Note 4**).

**Péclet number of particles for estimating effect of Brownian motion and diffusion.** The standard description of the Langevin equation on particles includes external driving forces and random Brownian force (*ξ*(*t*)). When the advective and diffusive transportation of suspended particles occur simultaneously, the motion of moving particles in the fluid can be presumed using Péclet number for mass transfer (Pe), defined as the ratio of transport rate of advection to diffusion. A system with Pe >> 1 governs the movement of suspended particles depending on the external driving forces (ACEO or DEP for our case), while the motions of particles are dominated by the Brownian diffusion for Pe << 1. Utilizing the mass diffusion coefficient (*D*) from Stokes–Einstein relationship where *D* = *k*_B_*T*/6π*ηR* (*k*_B_ for Boltzmann coefficient), Pe for mass transfer is given by

Pe = *Lu*/*D* (10)

where *L* and *u* represent characteristic length of the system (in the order of ~10 μm) and fluid velocity, respectively. Since the simulated velocities were typically in the order of ~10^-4^ m·s^-1^ (**Supplementary Note 4** and **Main Fig. 3**), Pe of PS particles was calculated to be Pe >> 1 for most of the cases; Pe ≈ 2 × 10^3^, 4 × 10^2^, 2 × 10^2^, and 1 × 10^2^ for PS particles with its diameters of 1 μm, 200 nm, 100 nm, and 50 nm, respectively. Therefore, we concluded that the effects of diffusion are negligible relative to the driving force, thereby the stochastic term *ξ*(*t*) can be neglected from the Langevin equation[^23^](#_ENREF_23).

**Supplementary Note 6: Stokes numbers of particles.**

The Stokes number (Stk) is the dimensionless number that characterizes the particle behaviour suspended in a fluid flow, which is defined as the ratio of characteristic time of suspended particle to that of the fluid[^24^](#_ENREF_24)

Stk = 2*ρ_p_uR*^2^/9*ηL*. (11)

A particle with Stk >> 1 maintains its initial trajectory, without much deflection, instead of following the streamline of flow. On the contrary, for Stk << 1, the motion of the particles is dominated by the viscous force with negligible inertial effect, and therefore, is tightly coupled to the fluid streamlines. Since velocities arising from ACEO dynamics were calculated to be in the order of ~10^-4^ m·s^-1^ (**Supplementary Note 4** and **Main Fig. 3**), an Stk << 1 is calculated for the moving PS particles with various diameters used in our experiments. For example, Stk of PS particles with diameters of 1 μm, 200 nm, 100 nm, and 50 nm are Stk ≈ 7 × 10^-7^, 3 × 10^-8^, 7 × 10^-9^, and 2 × 10^-9^, respectively. Therefore, we conclude that suspended particles of our experiments move along the fluid streamlines, instead of detaching from the streamlines where the flow abruptly changes.

**Supplementary Note 7: Thermal characteristics of VNE.**

Apart from dielectrophoretic enhancement (**Main Fig. 1**), thermal Joule heating effect was also investigated to validate the viability of biomaterial in the presence of E-fields. For a reliable analysis and comparison between VNE and HNE, the materials of electrodes were set to be Au instead of using ITO at the bottom of the VNE. Further, other simulation conditions such as substrates, aqueous environment, cover slip (using physical values in **Supplementary Table 1**), and thickness of electrode (40 nm) were also maintained equally. In particular, two simulation models of VNE and HNE were strictly controlled to maintain identical meshing conditions that are crucial for comparing results. Temperature increment (Δ*T*) was simulated on both VNE and HNE for various values of *V*_pp_. According to the results, the voltage-dependent behavior of Δ*T* was considerably similar to that of the gradient of E-field squared (∇**E**^2^) as the maximum value of Δ*T* (Δ*T*_max_) was located at the edge of the electrodes and the relation between Δ*T*_max_ and *V*^2^_pp_ is linear; Δ*T*_max_ = *βV*^2^_pp_ (**Supplementary Fig. 5**). Further, the thermal enhancement on VNE with *d* = 100 nm was also comparable to HNE with *d* = 20 nm, which corresponds to the dielectrophoretic performance described in the main manuscript.


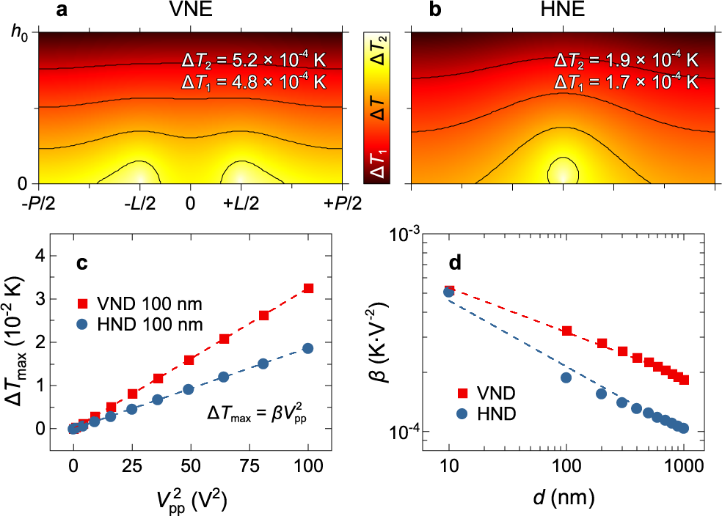


**Supplementary Figure** **5. Thermal characteristics of VNE and HNE.** **a**, **b** Calculated distribution maps of Δ*T* on VNE (**a**) and HNE (**b**) with gap distance of *d* = 100 nm under *V*_pp_ = 1 V. **c**, **d** Calculated Δ*T*_max_ on VNE (red square) and HNE (blue circle) as a function of *V*^2^_pp_ (**c**) and its corresponding *β* on VNE (red square) and HNE (blue circle) as a function of *d* (**d**).

**Supplementary Note 8: Additional simulation and numerical analysis of ACEO.**


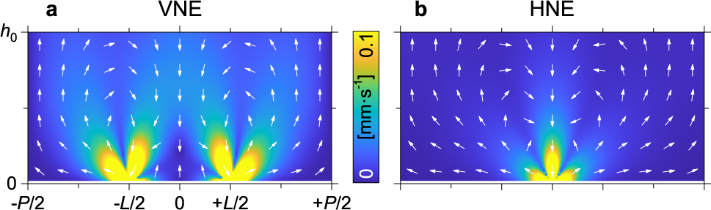


**Supplementary Figure** **6. ACEO-induced micro-vortices under 10 kHz.** **a**, **b** Calculated distribution of ACEO flow on VNE (**a**) and HNE (**b**) with a gap distance of *d* = 100 nm under *V*_pp_ = 1 V and *f* = 10 kHz.


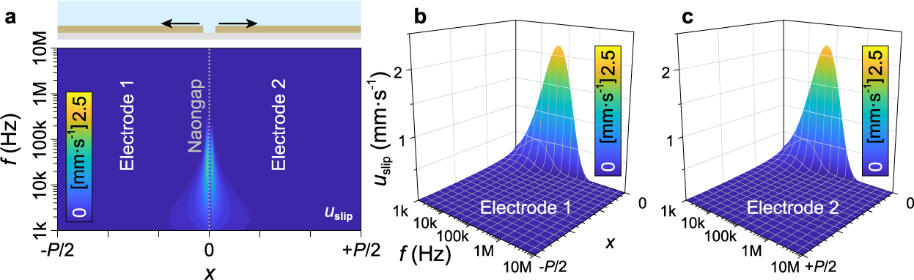


**Supplementary Figure** **7. Analysis of ACEO-induced slip velocity on HNE.** **a**-**c** Calculated slip velocity (*u*_slip_) distribution at the electrode surfaces of HNE (*d* = 100 nm) as a function of *f* (**a**, *V*_pp_ = 1 V) with schematic of *u*_slip_ (inset in **a**) and 3D illustrations of *u*_slip_ on electrode 1 (**b**) and 2 (**c**).

**Supplementary Note 9: Fabrication process of VNE array.**

A glass substrate (20 mm × 24 mm) with a 40-nm-thick patterned indium-tin-oxide (ITO) layer was sonicated in acetone and isopropyl alcohol for 10 min each and dried under a stream of N_2_ gas. A 4 *wt*.% poly(4-vinylphenol) (PVP) solution was prepared by dissolving PVP powder in a propylene glycol monomethyl ether acetate solvent with a thermal cross-linking agent, poly(melamine-co-formaldehyde) methylated (Sigma Aldrich). A 100-nm-thick PVP insulating film was prepared by spin-coating the mixed solution onto the glass surface at 3000 rpm for 30 s, followed by thermal annealing at 100 °C for 10 min and post-baking at 200 °C for a further 20 min to facilitate prompt cross-linking. For wiring procedures, the marginal area of the PVP insulator was partially removed by a sequential process of standard photolithography and oxygen (O_2_) plasma reactive ion etching (RIE). Prior to O_2_ plasma RIE, a photoresist, AZ1512 (MicroChemicals), was spin-coated onto the PVP film at 3000 rpm for 30 s and then annealed at 95 °C for 1 min. Subsequently, it was exposed to the i-line (wavelength of 365 nm) ultra-violet (UV) light of 25 mW through a photomask for 6 s using a mask aligner (MA-6 III, Karl-suss). The UV-exposed photoresist layer was developed in an AZ300 MIF developer (Merck) for 1 min. For removal of PVP from the electrodes, RIE (RIE 80 plus, Oxford Instrument) was carried out under a 100 sccm flow of O_2_ gas at a RF power of 150 W and a pressure of 0.1 Torr. After stripping the residual photoresist with acetone, a 40-nm-thick gold (Au) layer was thermally deposited (2.0 Å·s^-1^ at 10^-5^ Torr; MHS-1800, Muhan) on the PVP surface using a shadow mask. Periodic photoresist patterns consisting of arrays of 10-μm-diameter holes were prepared by additional photolithography processes, followed by 5 s of gold Au-etching (Gold Etchant TFA, Transene) and PVP etching (O_2_ plasma RIE for 30 s). Finally, the device was sonicated in acetone for 10 min to remove the residual photoresist. See **Supplementary Fig. 8** for a graphical illustration of the entire fabrication procedure.


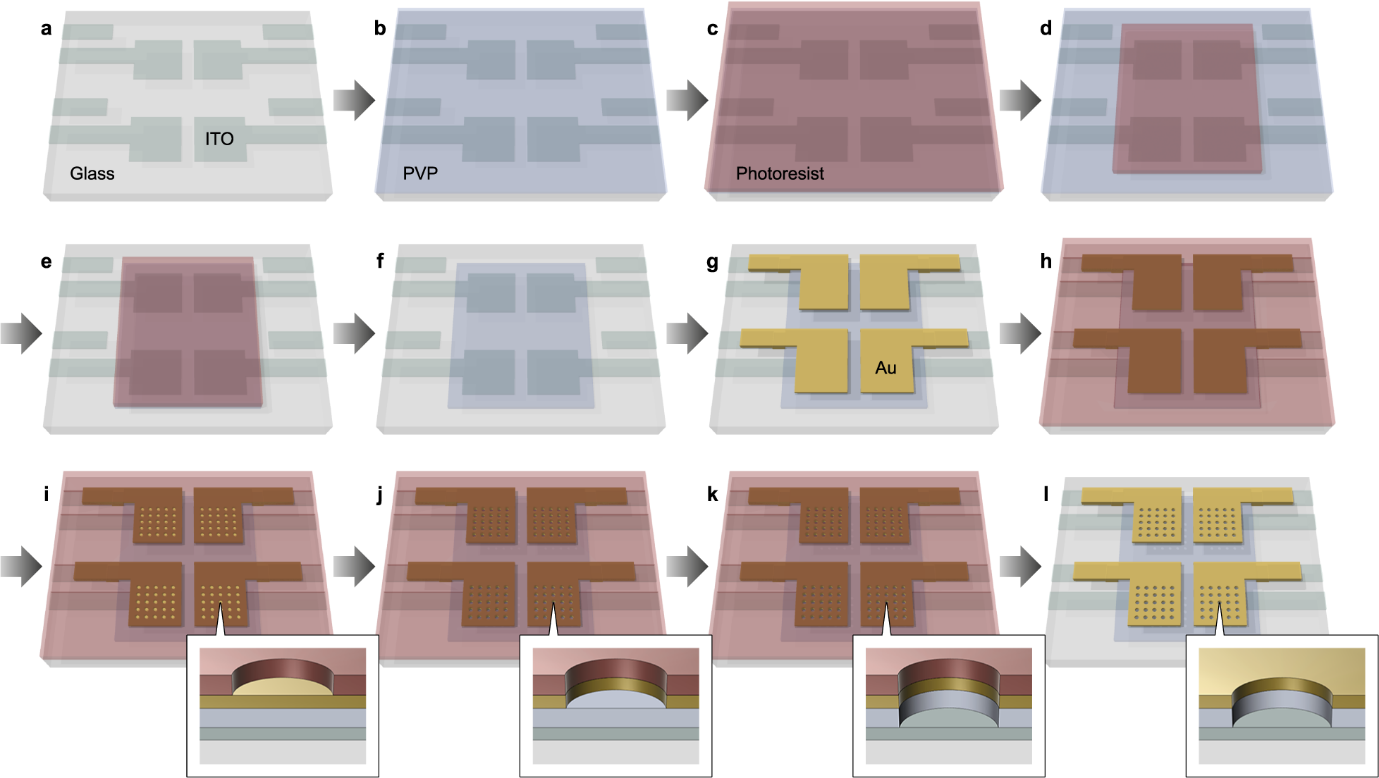


**Supplementary Figure** **8. Schematic of the detailed fabrication process.** **a** 40-nm-thick patterned ITO electrodes on a glass substrate. **b** Spin-coating of 100-nm-thick PVP layer as an insulator film. **c** Spin-coating of a photoresist layer. **d** Photolithographic patterning of photoresist layer into a rectangular pattern. **e** Removal of marginal area of PVP insulator by O_2_ plasma etching. **f** Removal of photoresist layer using acetone cleaning. **g** Thermal deposition of 40-nm-thick Au electrodes through shadow mask. **h** Spin-coating of additional photoresist layer. **i** Photolithographic patterning of photoresist patterns into micro hole array patterns. **j** Wet etching of Au electrode layer. **k** O_2_ plasma etching of PVP insulator layer. **l** Removal of photoresist layer using acetone.

**Supplementary Note** **10: Trapping of PS microparticles on VNE and voltage-dependent trapping capacity.**

**Preparation of PS microparticles.** Commercially available label-free 1 μm suspended polystyrene (PS) particles (4009A, Thermo Fisher Scientific) were used. To lower the electrical conductivity of the solution containing the suspended PS particles to 1 μS·cm^-1^, the solution was diluted by 1000 times using deionized (DI) water to a final concentration of 10 ppm (1.8 × 10^8^ particles·ml^-1^). A conductivity meter (LAQUAtwin EC-33, Horiba) was used for solution measurements.

**Calculation of CM factor of PS micro/nanoparticles.** The frequency-dependent CM factor, *f*_CM_(*ω*), can be calculated using the following equation:

$f_{CM}(\omega)=\frac{\varepsilon_{p}^{*}(\omega)-\varepsilon_{m}^{*}(\omega)}{\varepsilon_{p}^{*}(\omega)+2\varepsilon_{m}^{*}(\omega)}$ (12)

where *ε*_p_^*^(*ω*) and *ε*_m_^*^(*ω*) denote the complex permittivity of the particle and medium, respectively. Each complex permittivity is expressed as,

$\varepsilon_{p}^{*}(\omega)=\varepsilon_{p}^{*}-i\frac{\sigma_{p}}{\omega}$ and $\varepsilon_{p}^{*}(\omega)=\varepsilon_{p}^{*}-i\frac{\sigma_{p}}{\omega}$ (13)

in which *ε*_p_, *ε*_m_, *σ*_p_, *σ*_m_ are the permittivities and conductivities of the particle and medium, respectively (i = $\sqrt{\text{-1}}$). Equations (12) and (13) indicate that the polarizing behaviour is majorly governed by the material properties of the particles and medium conditions. Additionally, the local surface charge of the particles can be evaluated by adopting the Maxwell-Wagner-O’Konski (MWO) model, which is given by[^25^](#_ENREF_25),

*σ*_p_ = *σ*_bulk_ + 2*K*_surf_/*R* (14)

where *σ*_bulk_ and *K*_surf_ denote the bulk conductivity and surface conductance of the particles, respectively. Applying the parameters specified in **Supplementary Table 2** to equations (12-14), the real parts of the CM factor (Re[*f*_CM_(*ω*)]) were successfully calculated using their effective conductivities of *σ*_p_ = 114 μS·cm^-1^, 570 μS·cm^-1^, 1140 μS·cm^-1^, and 2280 μS·cm^-1^ for particles with diameters of 1 μm, 200 nm, 100 nm, and 50 nm, respectively (**Supplementary Fig. 9**).

| Parameters | Values |
| --- | --- |
| Radius of particles (*R*) | 0.5 μm, 100 nm, 50 nm, and 25 nm |
| Permittivity of PS (*ε*_p_) | 2.55·*ε*_0_ [[26](#_ENREF_26)] |
| Bulk conductivity of PS (*σ*_bulk_) | 10^-14^ S·m^-1^ [[27](#_ENREF_27)] |
| Surface conductance of PS (*K*_surf_) | 2.85 nS [[27](#_ENREF_27)] |
| Permittivity of medium (*ε*_m_) | 80·*ε*_0_ [[1](#_ENREF_1)] |
| Conductivity of medium (*σ*_m_) | 1 × 10^-4^ S·m^-1^ (Measured) |

**Supplementary Table** **2.** **Parameters for calculation of CM factor of PS micro/nanoparticles.**


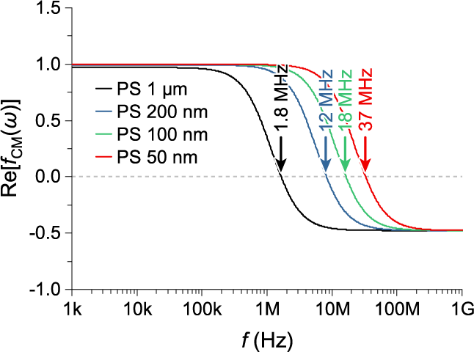


**Supplementary Figure** **9. Calculated CM factors of PS particles.** Calculated Re[*f*_CM_(*ω*)] of PS particles as a function of frequency; diameters of 1 μm (black), 200 nm (blue), 150 nm (green), and 50 nm (red). Note that corresponding crossover frequencies (*f*_χ_) of PS particles were estimated to be 1.8 MHz (1 μm), 12 MHz (200 nm), 18 MHz (100 nm), and 37 MHz (50 nm), respectively.

**Supplementary Note 11: Pseudo-potential for localization of particles.**

Particles suspended in an aqueous environment move and migrate to positions at which the potential energy is minimum. In this approach with non-conservative forces, the particle behaviour of pseudo-potential was introduced to interpret role of dissipative forces on localizing behaviours of particles. As two dominant forces affect the local position of the suspended particles on the VNE surface, the total pseudo-potential energy profile along the surface, *U*(*x*), can be given as *U*(*x*) = *U*_DEP_(*x*) + *U*_ACEO_(*x*), where *U*_DEP_ and *U*_ACEO_ indicate that the pseudo-potential energies arise from DEP- and ACEO driving forces. Note that two dominant pseudo-potential energies are achieved from the inverse gradient of **F**_DEP_ and **F**_ACEO_ using the equations *U*_DEP_ = -π*ε*_m_*R*^3^Re[*f*_CM_(*ω*)]**E**^2^ and **F**_ACEO_ = -∇*U*_ACEO_, respectively[^23^](#_ENREF_23)^,^[^28^](#_ENREF_28). Shown in **Supplementary Fig. 10**, the minimum value of *U*(*x*) is calculated at the centre of the VNE at *f* = 10 kHz (**Supplementary Fig. 10a**), in contrast to the edges of the VNE at *f* = 100 kHz (**Supplementary Fig. 10b**), respectively. This shows great consistency with the experimental demonstration shown in **Main Fig. 3**.


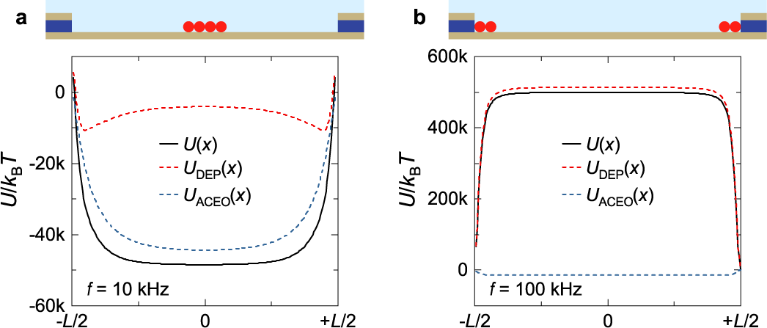


**Supplementary Figure** **10. Energy landscape of pseudo-potential on VNE surface.** **a**, **b** Calculated *U*_DEP_(*x*) (red), *U*_ACEO_(*x*) (blue), and *U*(*x*) (black) along the bottom electrode surface of VNE (*d* = 100 nm) under *V*_pp_ = 2.5 V, *f* = 10 kHz (**a**), and 100 kHz (**b**).

**Supplementary Note 12: Voltage-dependent trapping capacity on VNE.**


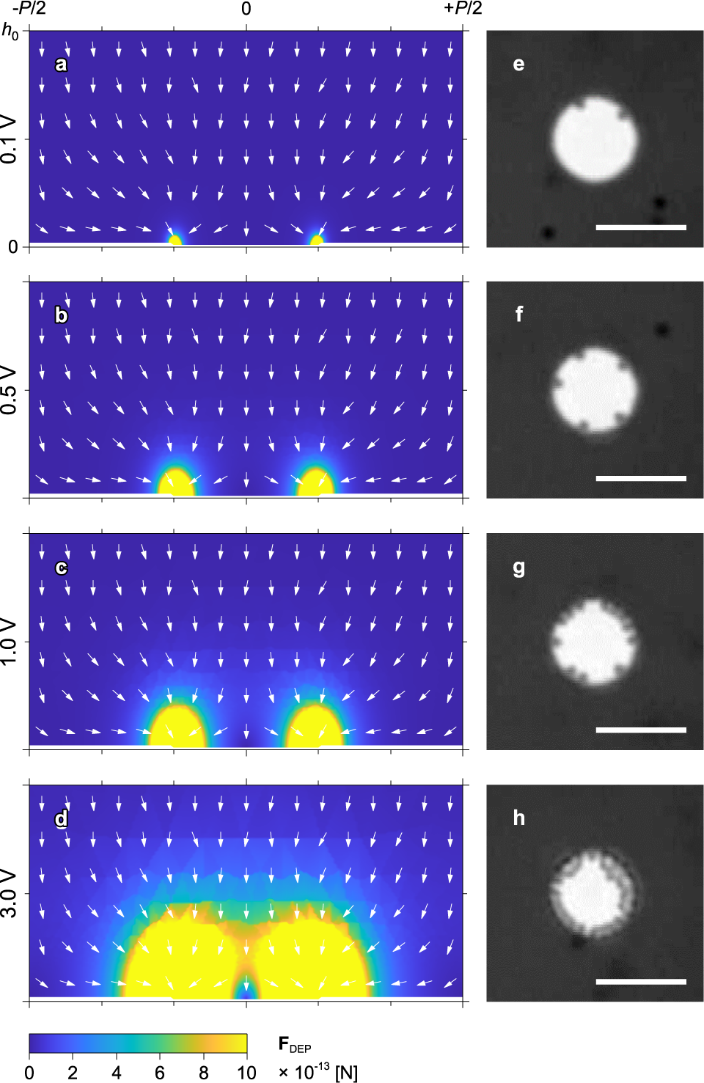


**Supplementary Figure** **11. Voltage-dependent particle trapping of VNE.** **a**-**h** Cross-sectional simulations of **F**_DEP_ (**a**-**d**) and top-view experimental BF micrographs (**e**-**h**) of suspended 1 μm PS particles on a single VNE under various AC voltages; *f* = 100 kHz, *V*_pp_ = 0.1 V (**a**, **e**), 0.5 V (**b**, **f**), 1.0 V (**c**, **g**), and 3.0 V (**d**, **h**). Note that each experiment was conducted for an identical voltage application duration of 20 s, and simple voltage boundary conditions were utilized for simulation. This confirms that higher voltage results in intensive force generation, and thus, improves the effective trapping volume. Scale bars: 10 μm.


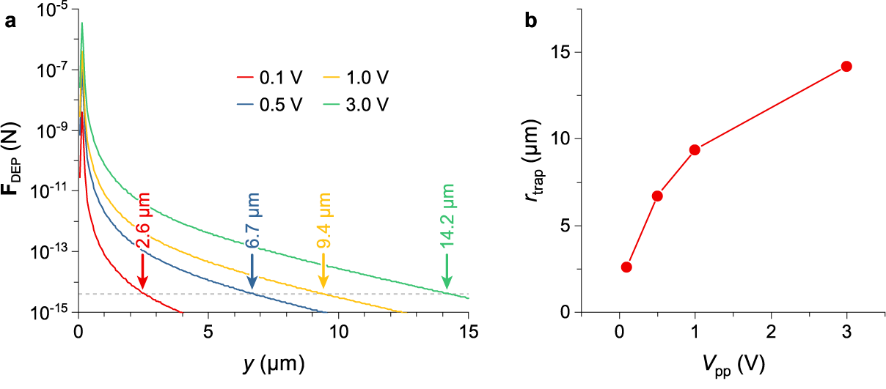


**Supplementary Figure** **12. Trapping radius of VNE on 1 μm PS particles.** **a** Calculated **F**_DEP_ (reproduced from **Supplementary Fig. 11**) of suspended 1 μm PS particles as a function of y-axial distance from the site of the maximum **F**_DEP_ (*y*). Note that calculated Brownian thermal force (**F**_th_) is represented by a grey dotted line. AC voltage variation at constant values of frequency of *f* = 100 kHz confirms that the effective trapping radius increases as applied voltage increases; *V*_pp_ = 0.1 V (red), 0.5 V (blue), 1.0 V (yellow), and 3.0 V (green). Arrows indicate the point at which **F**_DEP_ exceeds **F**_thermal_ (**F**_DEP_ = **F**_thermal_), which represents effective trapping radii (*r*_trap_). **b** Calculated *r*_trap_ of 1 μm PS particles as a function of applied AC voltage amplitudes.

**Supplementary Note 13: Trapping of yeast on VNE.**

**Preparation of yeast cells.** To prepare the yeast cells, *Yarrowia lipolytica* ATCC MYA-2613 strain was pre-inoculated into yeast synthetic complete (YSC) medium containing 6.7 g·L^-1^ of yeast nitrogen base (YNB), 20 g·L^-1^ of glucose, and complete supplement mixture (MP Biomedicals, Solon, OH, USA), and cultivated for 24 h at 28 ºC. The cells were then inoculated into a 250 ml flask containing 25 ml of the respective medium, and cultivated at 28 ºC for 144 h with orbital shaking at 200 rpm. The elongated cells were prepared by exposing spherical cells to stress condition with DI water for 3 h.

**Calculation of CM factor of yeast Cells.** To simplify the complexity of the biomaterial structure, the multi-shell model was adopted to evaluate the effective dielectric response of spherical yeast cells[^29-31^](#_ENREF_29). Based on the two-shell model consisting of three distinct regions of highly conductive cytoplasm, ultrathin lossy lipid membrane, and cell wall (**Supplementary Fig. 13a**), the CM factor is calculated using homogeneous effective permittivity of the model. The CM factor is given as follows,

$\varepsilon_{p}^{*}(\omega)=\varepsilon_{w}^{*}(\omega)\frac{\left(R_{3}/R_{2}\right)^{3}+2\left[\left\{\varepsilon_{2}^{*}(\omega)-\varepsilon_{w}^{*}(\omega)\right\}/\left\{\varepsilon_{2}^{*}(\omega)+2\varepsilon_{w}^{*}(\omega)\right\}\right]}{\left(R_{3}/R_{2}\right)^{3}-\left[\left\{\varepsilon_{2}^{*}(\omega)-\varepsilon_{w}^{*}(\omega)\right\}/\left\{\varepsilon_{2}^{*}(\omega)+2\varepsilon_{w}^{*}(\omega)\right\}\right]}$ (15)

and the parameters are given by,

$\varepsilon_{2}^{*}(\omega)=\varepsilon_{LB}^{*}(\omega)\frac{\left(R_{2}/R_{1}\right)^{3}+2\left[\left\{\varepsilon_{c}^{*}(\omega)-\varepsilon_{LB}^{*}(\omega)\right\}/\left\{\varepsilon_{c}^{*}(\omega)+2\varepsilon_{LB}^{*}(\omega)\right\}\right]}{\left(R_{2}/R_{1}\right)^{3}-\left[\left\{\varepsilon_{c}^{*}(\omega)-\varepsilon_{LB}^{*}(\omega)\right\}/\left\{\varepsilon_{c}^{*}(\omega)+2\varepsilon_{LB}^{*}(\omega)\right\}\right]}$, (16)

*ε*_w_^*^(*ω*) = *ε*_w_ – *j*(*σ*_w_/*ω*), *ε*_LB_^*^(*ω*) = *ε*_LB_ – *j*(*σ*_LB_/*ω*), *ε*_c_^*^(*ω*) = *ε*_c_ – *j*(*σ*_c_/*ω*) *R*_3_ = *R*, *R*_2_ = *R*_3_ - *t*_w_, and *R*_1_ = *R*_2_ - *t*_LB_, respectively.

However, for elongated yeast cells, the spheroidal shell model with a long axis radius of *R_x_* and short axis radii of *R_y_* = *R_z_* was utilized (**Supplementary Fig. 13b**)[^32^](#_ENREF_32)^,^[^33^](#_ENREF_33). In this model, the CM factor is given by[^33-35^](#_ENREF_33)

$f_{CM}(\omega)=\frac{\varepsilon_{p}^{*}(\omega)-\varepsilon_{m}^{*}(\omega)}{\left\{\varepsilon_{p}^{*}(\omega)-\varepsilon_{m}^{*}(\omega)\right\}A+\varepsilon_{m}^{*}(\omega)}$ (17)

in which *A* denotes the depolarizing factor. Assuming that the E-field is parallel to the major *x*–axis for the ellipsoid of *R_x_* > *R_y_* = *R_z_*, *A* is given by[^34^](#_ENREF_34)^,^[^35^](#_ENREF_35)

$A=-\frac{R_{y}^{2}}{2R_{x}^{2}e^{3}}\left(2e-\ln\frac{1+e}{1-e}\right)$ (18)

where *e* = [1 - (*R_y_*/*R_x_*)^2^]^1/2^. While Re[*f*_CM_(*ω*)] in the spherical model is bounded within the range of -0.5 < Re[*f*_CM_(*ω*)] < 1, that of the spheroid model presents much greater Re[*f*_CM_(*ω*)] values, indicating that the DEP force acting on the elongated particle is much stronger than that on the spherical ones (**Supplementary Fig. 13c**). Since the attractive DEP force is stronger in the direction of the long-axis of spheroid, most elongated yeasts cells are aligned in the radial direction (**Supplementary Fig. 14**).

| Parameters | Values |
| --- | --- |
| Radius of yeast cell sphere (*R*) | 3 μm (Measured) |
| Thickness of yeast lipid membrane (*t*_LB_) | 8 nm [[36](#_ENREF_36)] |
| Thickness of yeast cell wall (*t*_w_) | 220 nm [[36](#_ENREF_36)] |
| Long axis radius of yeast cell spheroid (*R_x_*) | 5 μm (Measured) |
| Short axis radii of yeast cell spheroid (*R_y_* = *R_z_*) | 2 μm (Measured) |
| Permittivity of cytoplasm (*ε*_c_) | 50·*ε*_0_ [[36](#_ENREF_36)] |
| Conductivity of cytoplasm (*σ*_c_) | 0.2 S·m^-1^ [[36](#_ENREF_36)] |
| Permittivity of yeast lipid membrane (*ε*_LB_) | 6·*ε*_0_ [[36](#_ENREF_36)] |
| Conductivity of yeast lipid membrane (*σ*_LB_) | 2.5 × 10^-7^ S·m^-1^ [[36](#_ENREF_36)] |
| Permittivity of yeast cell wall (*ε*_w_) | 60·*ε*_0_ [[36](#_ENREF_36)] |
| Conductivity of yeast cell wall (*σ*_w_) | 1.4 × 10^-2^ S·m^-1^ [[36](#_ENREF_36)] |
| Permittivity of outer medium (*ε*_m_) | 80·*ε*_0_ [[1](#_ENREF_1)] |
| Conductivity of outer medium (*σ*_m_) | 1 × 10^-4^ S·m^-1^ (Measured) |

**Supplementary Table 3.** **Parameters for calculation of CM factor of yeast cells.**


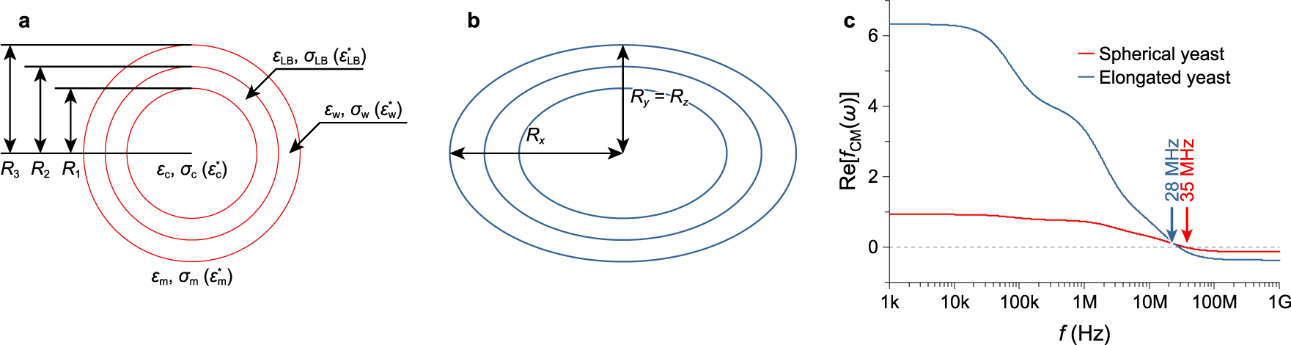


**Supplementary Figure** **13.** **Dielectric model and calculated CM factor of yeast cells.** **a**-**c** Schematic illustrations of dielectric models of the yeast cell based on the two-shell model (**a**), and the ellipsoid shell model (**b**). Calculated Re[*f*_CM_(*ω*)] of spherical- (red) and elongated yeast cells (blue) as a function of frequency (**c**). Note that *f*_χ_ values of spherical and elongated yeast cells were estimated to be 35 MHz and 28 MHz, respectively.


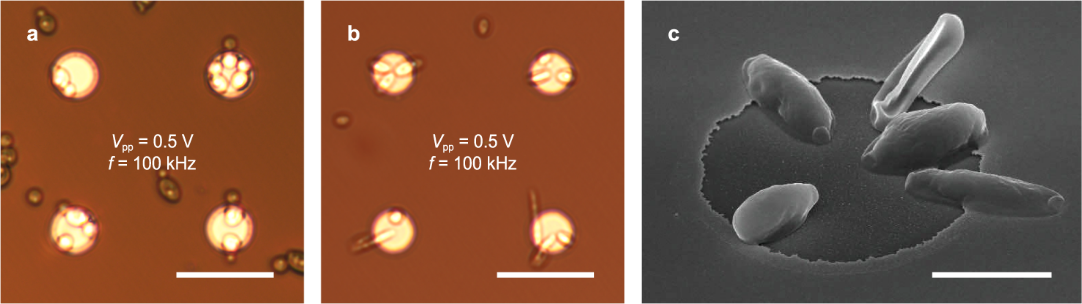


**Supplementary Figure** **14. Trapping of yeast cells on VNE.** **a**, **b** BF microscopic images of spherical- (**a**) and elongated yeast cells (**b**) trapped at the edge of the VNE electrodes under AC frequencies of *V*_pp_ = 0.8 V and *f* = 100 kHz. Scale bars: 20 μm. **c** SEM image of trapped elongated yeast cells. Scale bar: 5 μm.

**Supplementary Note 14: Trapping of *B*. *subtilis* spores on VNE.**

**Preparation of *Bacillus subtilis* spores.** To prepare the bacterial endospores, the nutrient exhaustion method reported by Foerster and Foster was adopted[^37^](#_ENREF_37). Specifically, *Bacillus subtilis* ATCC 6051 strain was pre-inoculated into a Luria-Bertani (LB) medium and grown overnight at 37 ºC. The cells were then inoculated into a 250 ml Erlenmeyer flask containing 20 ml of F growth medium (composed of 1 % glucose, 0.1 % L-glutamate, 0.05 % yeast extract, 0.5 % KH_2_PO_4_, 0.1 % (NH_4_)_3_PO_4_, 0.02 % MgSO_4_, 0.01 % NaCl, 0.005 % CaCl_2_, 0.0007 % MnSO_4_, 0.001 % ZnSO_4_, and 0.001 % FeSO_4_^)^[^38^](#_ENREF_38), and cultivated at 37 ºC for 72 h with orbital shaking at 200 rpm. The sporulated *B. subtilis* was stained for visualization using a Schaeffer and Fulton Spore Stain Kit (Sigma Aldrich, St. Louis, MO) according to the manufacturer’s instructions.

**Calculation of CM factor of *B. subtilis*.** The CM factor of *B*. *subtilis* was calculated using MWO theory, simplifying the complexity of the biomaterial structure into a homogeneous dielectric solid (**Supplementary Fig. 15**). Note that surface conductance was assumed from those of virus particles[^39-41^](#_ENREF_39).

| Parameters | Values |
| --- | --- |
| Radius of particles (*R*) | 400 nm (Measured) |
| Permittivity of *B*. *subtilis* (*ε*_p_) | 77·*ε*_0_ [[42](#_ENREF_42)] |
| Bulk conductivity of *B*. *subtilis* (*σ*_bulk_) | 9.35 × 10^-2^ S·m^-1^ [[43](#_ENREF_43)] |
| Surface conductance of *B*. *subtilis* (*K*_surf_) | 1 nS (Assumed from [[39-41](#_ENREF_39)]) |
| Permittivity of medium (*ε*_m_) | 80·*ε*_0_ [[1](#_ENREF_1)] |
| Conductivity of medium (*σ*_m_) | 1 × 10^-4^ S·m^-1^ (Measured) |

**Supplementary Table 4.** **Parameters for calculation of CM factor of *B*. *subtilis*.**


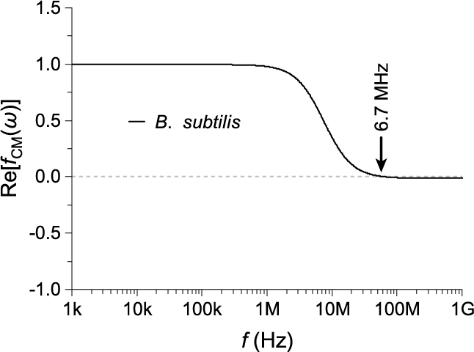


**Supplementary Figure** **15. Calculated CM factors of B. subtilis.** Calculated Re[*f*_CM_(*ω*)] of *B. subtilis* as a function of frequency. The *f*_χ_ of *B*. *subtilis* was estimated to be 6.7 MHz.


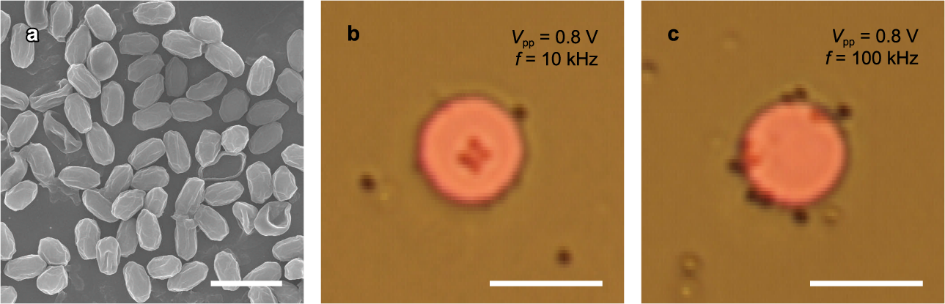


**Supplementary Figure** **16. Trapping of *B*. *subtilis* spores on VNE.** **a** SEM image of *B*. *subtilis* spores (~ 1 μm). Scale bar: 2 μm. **b**, **c** BF microscopic images of their trapping at the centre (**b**) and edge (**c**) of the VNE electrodes under AC frequencies of *f* = 10 kHz and 100 kHz, respectively (*V*_pp_ = 0.8 V). Scale bars: 10 μm.

**Supplementary Note 15: Trapping of PS nanoparticles on VNE.**

**Preparation of PS nanoparticles.** Commercially available suspended PS nanoparticles with fluorescently (FL) labelled 200 nm (blue; Ex/Em: 365 nm/412 nm), 100 nm (green; Ex/Em: 468 nm/508 nm), and 50 nm (red; Ex/Em: 542 nm/612 nm; Fluoro-Max series, Thermo Fisher Scientific) nanoparticles were used. The three different particles solutions are mixed into a single mixture. To lower the electrical conductivity of the mixture solution to 1 μS·cm^-1^, the solution was diluted by 1000 times using DI water to its final total concentration of 10 ppm and respective concentrations of 7.7 × 10^8^, 6.1 × 10^9^, and 4.9 × 10^10^ particles·ml^-1^ for 200, 100, and 50 nm PS particles.


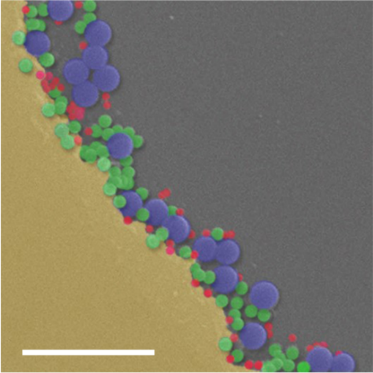


**Supplementary Figure** **17. Trapping of PS nanoparticles on VNE.** SEM image of PS nanoparticles with their diameters of 200 nm (blue), 100 nm (green), and 50 nm (red) trapped at the edge of VNE electrode (yellow) after the selective trapping process. Note that the image was pseudo-coloured for ease of observation. Scale bar: 1 μm.

**Supplementary Note 16: Trapping of SUVs on VNE.**

**Preparation of SUVs (DOPC:NBD- PC = 97:3).** Lipid molecules labelled with green FL dyes, 1-oleoyl-2-{6-[(7-nitro-2-1,3-benzoxadiazol-4-yl)amino]hexanoyl}-sn-glycero-3-phosphocholine (NBD-PC) and 1,2-dioleoyl-sn-glycero-3-phosphocholine (DOPC), were dissolved at a molecular ratio of 3:97 in chloroform (Sigma Aldrich) at 0.1 mg·ml^-1^. All lipids were purchased from Avanti Polar Lipids (Alabaster, AL, USA). The rapid solvent exchange method[^44^](#_ENREF_44) was employed to exchange the chloroform in DI water, resulting in spherical-shaped lipid balls in the form of the multi-lamellar vesicles (MLVs). To normalize the size and lipid structures to 50-nm-diameter small unilamellar vesicles (SUVs), the solution was extruded 20 times through polycarbonate membranes with a pore size of 50 nm. The SUV mixture was then diluted by 100 times using DI water to the final vesicle concentration of 62.5 pM.

**Calculation of CM factor of SUV.** To evaluate induced dipole polarization of SUVs, the walled cell model of Jones, which consists of three different regions of cell interior, cell membrane and the wall, was adopted (**Supplementary Fig. 18a**)[^45^](#_ENREF_45). By replacing the wall and cytoplasm of the model with a lossy lipid membrane shell of finite thickness and an aqueous medium, respectively, the complex permittivity of the dielectric model (*ε*_p_^*^(*ω*)) is given by,

$\varepsilon_{p}^{*}(\omega)=\varepsilon_{w}^{*}(\omega)\frac{\left(R_{2}/R_{1}\right)^{3}+2\left[\left\{\varepsilon_{c}^{*}(\omega)-\varepsilon_{LB}^{*}(\omega)\right\}/\left\{\varepsilon_{c}^{*}(\omega)+2\varepsilon_{LB}^{*}(\omega)\right\}\right]}{\left(R_{2}/R_{1}\right)^{3}-\left[\left\{\varepsilon_{c}^{*}(\omega)-\varepsilon_{LB}^{*}(\omega)\right\}/\left\{\varepsilon_{c}^{*}(\omega)+2\varepsilon_{LB}^{*}(\omega)\right\}\right]}$ (19)

where each parameter is defined as, *ε*_im_^*^(*ω*) = *ε*_im_ – *j*(*σ*_im_/*ω*), *ε*_LB_^*^(*ω*) = *ε*_LB_ – *j*(*σ*_LB_/*ω*), and

$\varepsilon_{c}^{*}(\omega)=\frac{c_{LB}R_{1}\varepsilon_{im}^{*}(\omega)}{c_{LB}R_{1}+\varepsilon_{im}^{*}(\omega)}$ (20)

with their values specified in **Supplementary Table 5**. By substituting *ε*_p_^*^(*ω*) of equation (12) into that of equation (19), the CM factor of SUV was predicted as a function of the applied frequency (**Supplementary Fig. 18b**).

| Parameters | Values |
| --- | --- |
| Radius of SUV sphere (*R*_2_) | 25 nm |
| Thickness of lipid membrane bilayer (*t*_LB_) | 5 nm [[46-50](#_ENREF_46)] |
| Radius of inner medium (*R*_1_ = *R*_2_ – *t*_LB_) | 20 nm |
| Permittivity of inner medium (*ε*_im_) | 80·*ε*_0_ [[1](#_ENREF_1)] |
| Conductivity of inner medium (*σ*_im_) | 1 × 10^-4^ S·m^-1^ (Measured) |
| Permittivity of DOPC lipid membrane bilayer (*ε*_LB_) | 2.2·*ε*_0_ [[51](#_ENREF_51),[52](#_ENREF_52)] |
| Conductivity of DOPC lipid membrane bilayer (*σ*_LB_) | 9.7 × 10^-3^ S·m^-1^ [[51](#_ENREF_51),[53](#_ENREF_53)] |
| Capacitance of DOPC lipid membrane bilayer (*c*_LB_) | 1.85 × 10^-2^ F·m^-2^ [[51](#_ENREF_51),[54](#_ENREF_54)] |
| Permittivity of outer medium (*ε*_m_) | 80·*ε*_0_ [[1](#_ENREF_1)] |
| Conductivity of outer medium (*σ*_m_) | 1× 10^-4^ S·m^-1^ (Measured) |

**Supplementary Table** **5.** **Parameters for calculation of CM factor of SUV.**


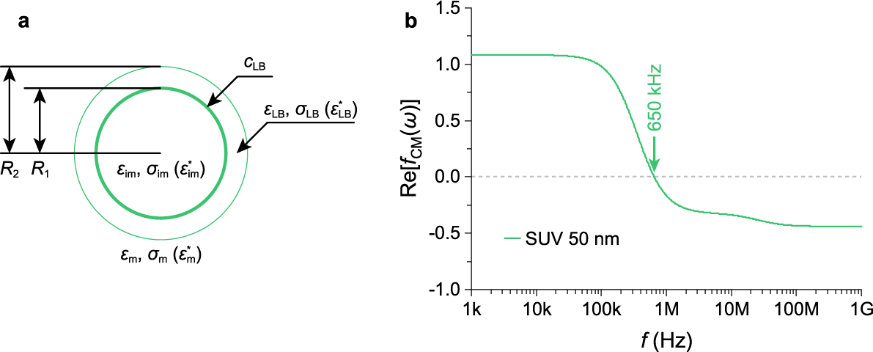


**Supplementary Figure 18. Dielectric model and calculated CM factor of SUV.** **a**, **b** Schematic illustration of the dielectric model of SUV based on the walled cell model (**a**; Reproduced from [[45](#_ENREF_45)]) and calculated Re[*f*_CM_(*ω*)] of SUV as a function of frequency (**b**). The *f*_χ_ of SUV was estimated to be 650 kHz.

**Supplementary Note 17: Large-area FL analysis for high-throughput and uniform trapping on VNE.**

To evaluate the large-area performance, FL microscopic images of captured SUVs on an array of *N_x_* × *N_y_* VNE unit cells were analyzed through a numerical analysis (Matlab, Mathworks). The FL images were prepared in the presence of applied AC voltages of *f* = 10 and 100 kHz (*V*_pp_ = 2 V shown in **Main Figs. 5b** and **5c**) showing trapping of 50 nm SUV on 20 × 20 multi-unit cells (total number of n = 400) over a large area (0.8 mm × 0.8 mm) without any false electrode. For total pixel numbers of *N_x_X* × *N_y_Y* (*X* and *Y* are pixel periodicities along *x*- and *y*-axis), the FL distribution of the images can be expressed as *I*(*x*, *y*) with *x* and *y* in the ranges of 0 < *x* < *N_x_X* and 0 < *y* < *N_y_Y*. Note that *x* and *y* are integers that represent the *x*- and *y*-coordinates of the pixel positions. Then, the distribution of the FL intensity achieved from a single cell in the *m*-th column and *n*-th row can be expressed as *I*_(_*_m_*_,_*_n_*_)_(*x*, *y*) = *I*(*x* + *X*(*m* - 1), *y* + *Y*(*n* - 1)) where 0 < *x* < *X* and 0 < *y* < *Y* (**Supplementary Fig. 19a**; Step 1). By carrying out image-processing and numerical analysis (**Supplementary Fig. 19a**; Step 2), the average FL distribution (*I*_avg_(*x*, *y*)) was calculated from every *I*_(_*_m_*_,_*_n_*_)_(*x*, *y*), as follows

$I_{avg}(x,y)=\sum_{m}^{N_{x}} \sum_{n}^{N_{y}} I_{(m,n)}(x,y)/N_{x}N_{y}$. (21)

Subsequently, by summing the FL intensities of all pixels from *I*_avg_(*x*,*y*) and dividing them by the total pixel number of the unit cell, the average FL intensity of *I*_avg_(*x*,*y*) (*I*_avg_) was calculated (**Supplementary Fig. 19a**; Step 3). It is given by,

$I_{avg}=\sum_{x}^{X} \sum_{y}^{Y} I_{avg}(x,y)/XY$. (22)

For minimum and maximum FL intensities of 0 and 255 (arbitrary unit), *I*_avg_ values were calculated to be 66 and 57 for *f* = 10 and 100 kHz, respectively (**Supplementary Figs. 19b** and **19c**). After obtaining average FL intensities of each unit cell (*I*_(_*_m_*_,_*_n_*_)_) as follows (**Supplementary Fig. 19a**; Step 4),

$I_{(m,n)}=\sum_{x}^{X} \sum_{y}^{Y} I_{(m,n)}(x,y)/XY$ (23)

normalized FL intensities of each unit cell (*I*^*^_(_*_m_*_,_*_n_*_)_) were calculated by dividing each *I*_(_*_m_*_,_*_n_*_)_ by *I*_avg_ (**Supplementary Fig. 19a**; Steps 5 and 6),

*I*^*^_(_*_m_*_,_*_n_*_)_ = *I*_(_*_m_*_,_*_n_*_)_/*I*_avg_, (24)

over 20 × 20 array (**Supplementary Figs. 19d** and **19e**). The standard deviations (s. d.) of 0.054 (*f* = 10 kHz) and 0.052 (*f* = 100 kHz) indicate that 90 % of the unit cells exhibit variations in FL intensity within ± 10 %, demonstrating the large-area uniformity of the proposed VNE array (**Supplementary Figs. 19f** and **19g**).


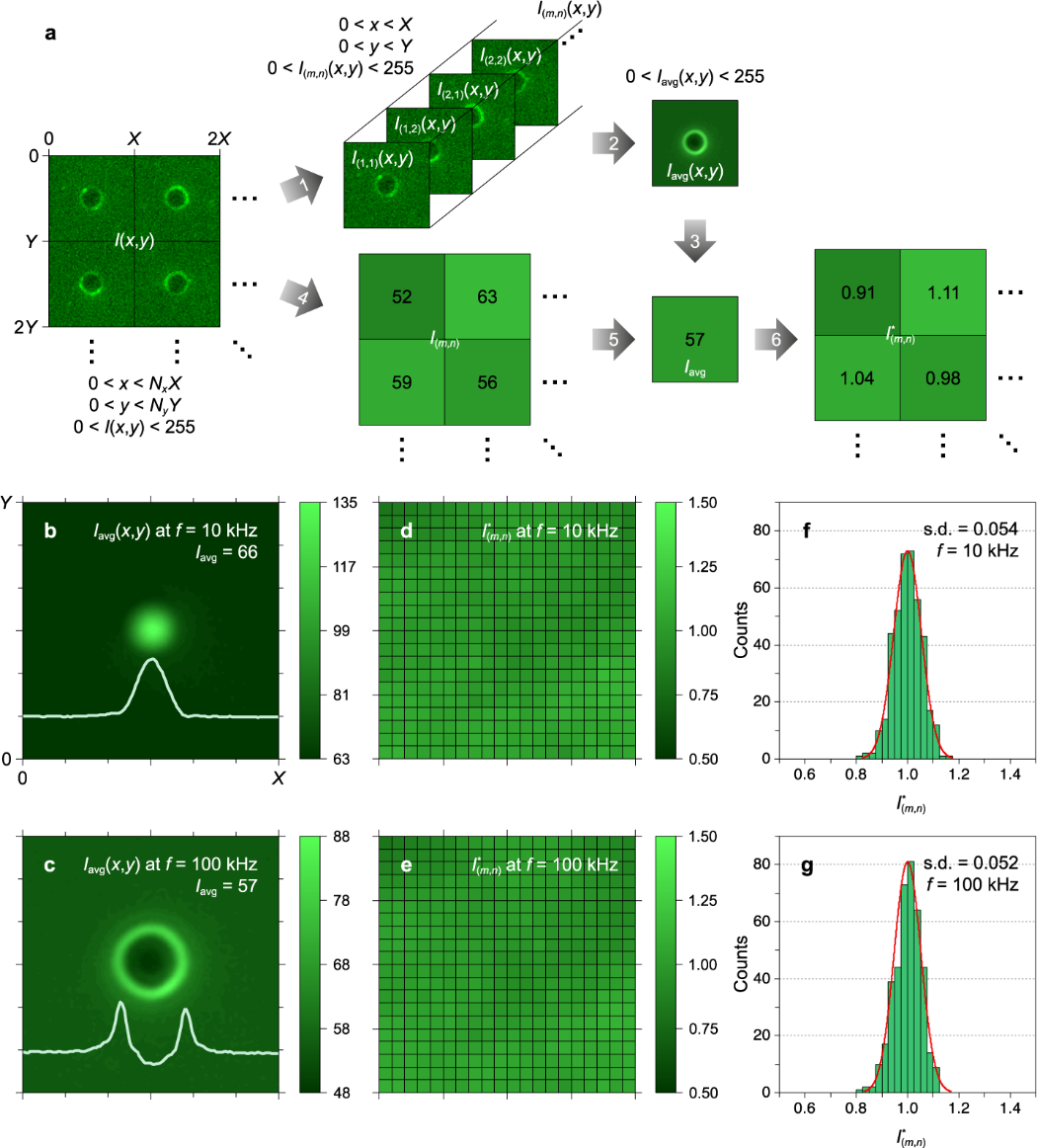


**Supplementary Figure** **19. Large-area FL analysis of SUV trapping on VNE. a** Schematics of image-processing and numerical analysis of FL micrographs. **b-g** FL analysis on 400 VNE unit cells capturing 50 nm-diameter SUVs at the centre (**b**, **d**, **f**, *f* = 10 kHz) and edge (**c**, **e**, **g**, *f* = 100 kHz) under *V*_pp_ = 2 V. Averaged FL distribution with its intensity and central FL intensity profiles (white lines) (**b**, **c**), normalized FL intensities on an array of 20 × 20 unit cells (**d**, **e**), and their FL histogram graphs (**f**, **g**).

**Supplementary Note 18: Trapping of Aβ_42_ fibrils on VNE.**

**Preparation of Aβ_42_ assemblies.** For the solubilization of lyophilized‐amyloid β‐protein (Aβ_42_) (Bachem AG, Bubendorf, Switzerland), we used 100 % dimethyl sulfoxide (Sigma, Saint Louis, MO, USA) to make a stock solution of 1 mM concentration. We added 20 μL of the Aβ_42_ solution to 200 μL of DI water and then incubated the solution (100 μM) at 37 ºC for 4 h and 24 h, separately. After incubation, a 50 μM thioflavin-T solution was added to stain the assembly structure of Aβ_42_. The unbound thioflavin-T was removed using an ultra-centrifugal filter (3000 molecular weight cut-off; Amicon, Merck Millipore). For AC electrokinetic trapping, stained Aβ_42_ was diluted by 10000 times using DI water, resulting in a final Aβ_42_ concentration of 1 nM and an electrical solution conductivity of 1 μS·cm^-1^.

**Calculation of CM factor of Aβ_42_ assemblies.** To achieve dielectric properties of Aβ_42_ fibrils, an ellipsoidal approximation was employed to simplify the high aspect ratio of the fibrous structure into an ellipsoidal model (**Supplementary Fig. 20a**)[^55^](#_ENREF_55). Using equations (17) and (18), the Re[*f*_CM_(*ω*)] of the ellipsoidal Aβ_42_ fibrils (**Supplementary Fig. 20b**) was estimated with a depolarizing factor of *A* = 0.0047. In this process, the surface conductance of the Aβ_42_ fibrils was also considered by using the effective surface conductivity of a charged ellipsoidal particle when the long-axis is parallel to the E-field, which is given by[^56^](#_ENREF_56),

$\sigma_{p}=\sigma_{bulk}+\frac{3}{2R_{y}}K_{surf}\cosh\xi_{0}I_{1}$. (25)

in which *I*_1_ = (1 - sinh^2^*ξ*_0_)·arcsin(1/cosh*ξ*_0_) + sinh*ξ*_0_ and tanh*ξ*_0_ = *R_y_*/*R_x_*.

While CM factor of the Aβ_42_ fibrils was calculated by ellipsoidal approximation, the spherical dielectric model together with the MWO theory was employed for Aβ_42_ oligomers/protofibrils (**Supplementary Fig. 20b**). Owing to the lack of literature, we assumed the surface conductance of Aβ_42_ from those of biological macromolecules[^57^](#_ENREF_57). Although the DEP force is much stronger in the direction parallel to the long-axes of spheroids, the captured Aβ_42_ fibrils are observed to be aligned along the VNE edge. This is attributed to their greater structural flexibility (**Supplementary Fig. 21** and **Main Figs. 5f** and **5g**) compared with stiff yeast cells (**Supplementary Fig. 14**).

| Parameters | Values |
| --- | --- |
| Long axis radius of Aβ_42_ fibril spheroid (*R_x_*) | 1.25 μm (Measured) |
| Short axis radii of Aβ_42_ fibril spheroid (*R_y_* = *R_z_*) | 100 nm (Measured) |
| Radius of Aβ_42_ oligomer/protofibril sphere (*R*) | 30 nm (Measured) |
| Permittivity of Aβ_42_ protein (*ε*_p_) | 3·*ε*_0_ [[58](#_ENREF_58)] |
| Conductivity of Aβ_42_ protein (*σ*_p_) | 2.5 × 10^-6^ S·m^-1^ [[59](#_ENREF_59)] |
| Surface conductance of Aβ_42_ protein (*K*_surf_) | 10 nS (Assumed from [[57](#_ENREF_57)]) |
| Permittivity of outer medium (*ε*_m_) | 80·*ε*_0_ [[1](#_ENREF_1)] |
| Conductivity of outer medium (*σ*_m_) | 1 × 10^-4^ S·m^-1^ (Measured) |

**Supplementary Table** **6.** **Parameters for calculation of CM factor of Aβ_42_ assemblies.**


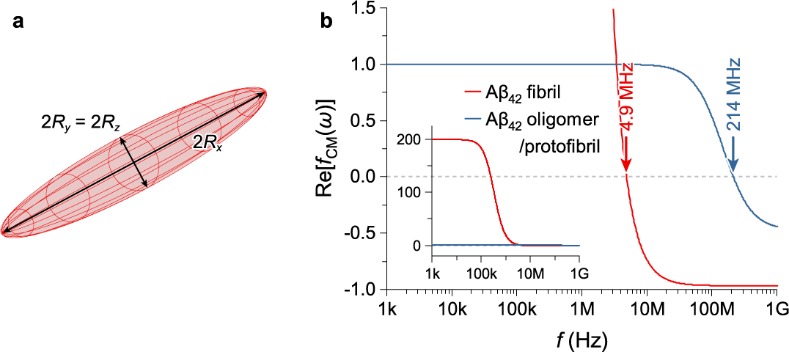


**Supplementary Figure 20.** **Dielectric model and calculated CM factor of Aβ_42_ assemblies.** **a**, **b** Schematic illustration of the dielectric model of Aβ_42_ fibrils based on the spheroidal dielectric model (**a**) and the calculated Re[*f*_CM_(*ω*)] of different types of Aβ_42_ assemblies; fibril (red) and oligomer/protofibril (blue) as a function of frequency (**b**). Note that the *f*_χ_ of the Aβ_42_ fibril and the oligomer/protofibril were estimated to be 4.9 MHz and 214 MHz, respectively.


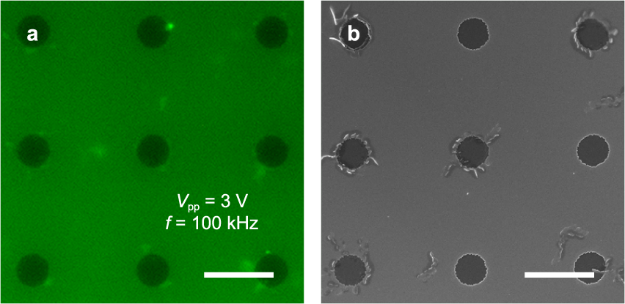


**Supplementary Figure** **21.** **Trapping of Aβ_42_ fibrils on VNE.** **a**, **b** FL microscopic- (**a**) and SEM image (**b**) of Aβ_42_ fibrils (24 h-incubated) trapped at the edge of VNE electrodes at an AC voltage of *f* = 100 kHz (*V*_pp_ = 3 V). Note that both images were captured for the same sample but from different spots. Scale bars: 20 μm.

**Supplementary Note 19: Characterization of Aβ_42_ assemblies.**

**CD spectrometer and DLS measurement.** After incubation of Aβ_42_ in DI water for 4 h, the Aβ_42_ structure was analysed by using a circular dichroism (CD) spectrometer (Chirascan plus, Applied Photophysics). We used a 1 mm path length cuvette and measured the signal range from 200 nm to 300 nm. Molecular structures of assembled peptide particles were estimated using the BeStSel program (Beta Structure Selection). The size of 4-h-incubated Aβ_42_ was estimated using the dynamic light scattering (DLS) (Zetasizer Nano, Malvern Instruments) technique. The Aβ_42_ proteins were filtered through a polycarbonate membrane with a pore diameter of 200 nm and then transferred to a DLS cuvette to perform the DLS measurement.


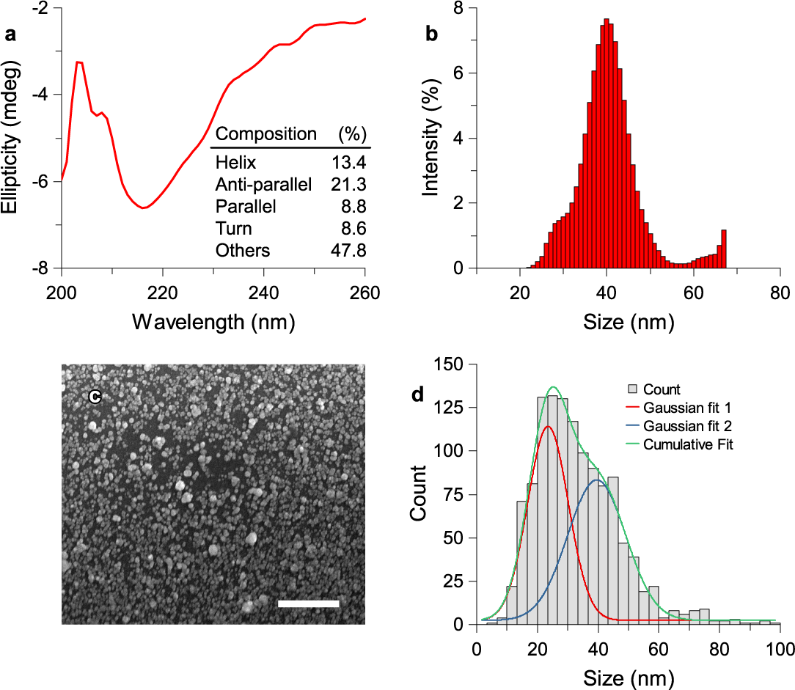


**Supplementary Figure** **22.** **Analysis of structure and size of Aβ_42_ proteins.** **a**-**d** CD spectrum (**a**; inset for structure of Aβ_42_ proteins), DLS data (**b**), SEM image (**c**), and corresponding SEM image analysis result (**d**) of Aβ_42_ proteins incubated for 4 h. Scale bar: 500 nm.

**Supplementary References**

1. Fernández, D. P., Mulev, Y., Goodwin, A. R. H. & Sengers, J. M. H. L. A database for the static dielectric constant of water and steam. *J. Phys. Chem. Ref. Data* **24,** 33-70 (1995).

2. Henninges, J., Huenges, E. & Burkhardt, H. In situ thermal conductivity of gas-hydrate-bearing sediments of the Mallik 5L-38 well. *J. Geophys. Res.: Solid Earth* **110,** B11206 (2005).

3. Tritt, T. M. *Thermal Conductivity: Theory, Properties, and Applications*. (Kluwer Academic/Plenum Publishers, New York, 2004).

4. Baeg, K. J., Noh, Y. Y., Ghim, J., Lim, B. & Kim, D. Y. Polarity effects of polymer gate electrets on non‐volatile organic field‐effect transistor memory. *Adv. Funct. Mater.* **18,** 3678-3685 (2008).

5. Kwon, Y.-W., Lee, C. H., Choi, D.-H. & Jin, J.-I. Materials science of DNA. *J. Mater. Chem.* **19,** 1353-1380 (2009).

6. Kim, G.-H. *et al.* High thermal conductivity in amorphous polymer blends by engineered interchain interactions. *Nat. Mater.* **14,** 295-300 (2015).

7. Alwan, E. A., Kiourti, A. & Volakis, J. L. Indium tin oxide film characterization at 0.1–20 GHz using coaxial probe method. *IEEE Access* **3,** 648-652 (2015).

8. Thuau, D., Koymen, I. & Cheung, R. A microstructure for thermal conductivity measurement of conductive thin films. *Microelectron. Eng.* **88,** 2408-2412 (2011).

9. Herth, E., Seok, S., Rolland, N. & Lasri, T. Wafer level packaging compatible with millimeter-wave antenna. *Sensor. Actuat. A: Phys.* **173,** 238-243 (2012).

10. Serway, R. A. & Jewett, J. W. *Principles of Physics*. (Saunders College Publishing, Fort Worth, 1998).

11. Ramos, A., Morgan, H., Green, N. G. & Castellanos, A. Ac electrokinetics: A review of forces in microelectrode structures. *J. Phys. D: Appl. Phys.* **31,** 2338-2353 (1998).

12. Ramos, A., Gonzalez, A., Castellanos, A., Green, N. G. & Morgan, H. Pumping of liquids with ac voltages applied to asymmetric pairs of microelectrodes. *Phys. Rev. E* **67,** 056302 (2003).

13. Loucaides, N., Ramos, A. & Georghiou, G. E. Novel systems for configurable AC electroosmotic pumping. *Microfluid. Nanofluid.* **3,** 709-714 (2007).

14. Green, N. G., Ramos, A. & Morgan, H. Numerical solution of the dielectrophoretic and travelling wave forces for interdigitated electrode arrays using the finite element method. *J. Electrostat.* **56,** 235-254 (2002).

15. Green, N. G., Ramos, A., Gonzalez, A., Castellanos, A. & Morgan, H. Electrothermally induced fluid flow on microelectrodes. *J. Electrostat.* **53,** 71-87 (2001).

16. Morgan, H. & Green, N. G. *AC Electrokinetics : Colloids and Nanoparticles*. (Research Studies Press, Baldock, 2003).

17. González, A., Ramos, A., Green, N. G., Castellanos, A. & Morgan, H. Fluid flow induced by nonuniform ac electric fields in electrolytes on microelectrodes. II. A linear double-layer analysis. *Phys. Rev. E* **61,** 4019-4028 (2000).

18. Green, N. G., Ramos, A., Gonzalez, A., Morgan, H. & Castellanos, A. Fluid flow induced by nonuniform ac electric fields in electrolytes on microelectrodes. III. Observation of streamlines and numerical simulation. *Phys. Rev. E* **66,** 026305 (2002).

19. Munson, B. R. *Fundamentals of Fluid Mechanics*. (John Wiley & Sons, Hoboken, 2013).

20. Chen, D. & Du, H. Simulation studies on electrothermal fluid flow induced in a dielectrophoretic microelectrode system. *J. Micromech. Microeng.* **16,** 2411-2419 (2006).

21. Lide, D. R. *CRC Handbook of Chemistry and Physics*. (CRC Press, Boca Raton, 2012).

22. Oh, J., Hart, R., Capurro, J. & Noh, H. M. Comprehensive analysis of particle motion under non-uniform AC electric fields in a microchannel. *Lab Chip* **9,** 62-78 (2009).

23. Juniper, M. P. N., Straube, A. V., Aarts, D. G. A. L. & Dullens, R. P. A. Colloidal particles driven across periodic optical-potential-energy landscapes. *Phys. Rev. E* **93,** 012608 (2016).

24. Liu, S.-J., Wei, H.-H., Hwang, S.-H. & Chang, H.-C. Dynamic particle trapping, release, and sorting by microvortices on a substrate. *Phys. Rev. E* **82,** 026308 (2010).

25. O'Konski, C. T. Electric properties of macromolecules. V. Theory of ionic polarization in polyelectrolytes. *J. Phys. Chem.* **64,** 605-619 (1960).

26. Green, N. G. & Morgan, H. Dielectrophoresis of submicrometer latex spheres. 1. Experimental results. *J. Phys. Chem. B* **103,** 41-50 (1999).

27. Honegger, T., Berton, K., Picard, E. & Peyrade, D. Determination of Clausius–Mossotti factors and surface capacitances for colloidal particles. *Appl. Phys. Lett.* **98,** 181906 (2011).

28. Pethig, R. R. *Dielectrophoresis: Theory, Methodology and Biological Applications*. (John Wiley & Sons, Hoboken, 2017).

29. Irimajiri, A., Hanai, T. & Inouye, A. A dielectric theory of “multi-stratified shell” model with its application to a lymphoma cell. *J. Theor. Biol.* **78,** 251-269 (1979).

30. Raicu, V., Raicu, G. & Turcu, G. Dielectric properties of yeast cells as simulated by the two-shell model. *Biochim. Biophys. Acta, Bioenerg.* **1274,** 143-148 (1996).

31. Turcu, I. & Lucaciu, C. M. Dielectrophoresis: A spherical shell model. *J. Phys. A: Math. Gen.* **22,** 985-993 (1989).

32. Asami, K., Hanai, T. & Koizumi, N. Dielectric approach to suspensions of ellipsoidal particles covered with a shell in particular reference to biological cells. *Jpn. J. Appl. Phys.* **19,** 359-365 (1980).

33. Kakutani, T., Shibatani, S. & Sugai, M. Electrorotation of non-spherical cells: Theory for ellipsoidal cells with an arbitrary number of shells. *Bioelectrochem. Bioenerg.* **31,** 131-145 (1993).

34. Morgan, H. & Green, N. G. Dielectrophoretic manipulation of rod-shaped viral particles. *J. Electrostat.* **42,** 279-293 (1997).

35. Stratton, J. A. *Electromagnetic Theory*. (McGraw-Hill, New York, 1941).

36. Patel, S. *et al.* Microfluidic separation of live and dead yeast cells using reservoir-based dielectrophoresis. *Biomicrofluidics* **6,** 34102-34102 (2012).

37. Tavares, M. B. *et al.* *Bacillus subtilis* endospores at high purity and recovery yields: Optimization of growth conditions and purification method. *Curr. Microbiol.* **66,** 279-285 (2013).

38. Foerster, H. F. & Foster, J. Endotrophic calcium, strontium, and barium spores of *Bacillus megaterium* and *Bacillus cereus*. *J. Bacteriol.* **91,** 1333-1345 (1966).

39. Hughes, M. P., Morgan, H. & Rixon, F. J. Dielectrophoretic manipulation and characterization of herpes simplex virus-1 capsids. *Eur. Biophys. J.* **30,** 268-272 (2001).

40. Hughes, M. P., Morgan, H. & Rixon, F. J. Measuring the dielectric properties of herpes simplex virus type 1 virions with dielectrophoresis. *Biochim. Biophys. Acta, Gen. Subj.* **1571,** 1-8 (2002).

41. Nakano, M., Ding, Z. & Suehiro, J. Dielectrophoresis and dielectrophoretic impedance detection of adenovirus and rotavirus. *Jpn. J. Appl. Phys.* **55,** 017001 (2015).

42. Kell, D. B. & Harris, C. M. On the dielectrically observable consequences of the diffusional motions of lipids and proteins in membranes. *Eur. Biophys. J.* **12,** 181-197 (1985).

43. Yu, A. C. S., Loo, J. F. C., Yu, S., Kong, S. K. & Chan, T.-F. Monitoring bacterial growth using tunable resistive pulse sensing with a pore-based technique. *Appl. Microbiol. Biotechnol.* **98,** 855-862 (2014).

44. Buboltz, J. T. & Feigenson, G. W. A novel strategy for the preparation of liposomes: Rapid solvent exchange. *Biochim. Biophys. Acta, Biomembranes* **1417,** 232-245 (1999).

45. Jones, T. B. *Electromechanics of Particles*. (Cambridge University Press, Cambridge, 1995).

46. Yoon, T.-Y. *et al.* Topographic control of lipid-raft reconstitution in model membranes. *Nat. Mater.* **5,** 281-285 (2006).

47. Ryu, Y.-S. *et al.* Reconstituting ring-rafts in bud-mimicking topography of model membranes. *Nat. Commun.* **5,** 4507 (2014).

48. Ryu, Y.-S. *et al.* Lipid membrane deformation accompanied by disk-to-ring shape transition of cholesterol-rich domains. *J. Am. Chem. Soc.* **137,** 8692-8695 (2015).

49. Ryu, Y.-S. *et al.* Curvature elasticity-driven leaflet asymmetry and interleaflet raft coupling in supported membranes. *Adv. Mater. Interfaces* **5,** 1801290 (2018).

50. Ryu, Y.-S. *et al.* Kinetics of lipid raft formation at lipid monolayer-bilayer junction probed by surface plasmon resonance. *Biosens. Bioelectron.* **142,** 111568 (2019).

51. Lim, J. K., Zhou, H. & Tilton, R. D. Liposome rupture and contents release over coplanar microelectrode arrays. *J. Colloid Interf. Sci.* **332,** 113-121 (2009).

52. Chan, K. L., Gascoyne, P. R. C., Becker, F. F. & Pethig, R. Electrorotation of liposomes: Verification of dielectric multi-shell model for cells. *Biochim. Biophys. Acta, Lipids Lipid Metab.* **1349,** 182-196 (1997).

53. Bakás, L., Chanturiya, A., Herlax, V. & Zimmerberg, J. Paradoxical lipid dependence of pores formed by the *Escherichia coli* α-hemolysin in planar phospholipid bilayer membranes. *Biophys. J.* **91,** 3748-3755 (2006).

54. Stauffer, V., Stoodley, R., Agak, J. O. & Bizzotto, D. Adsorption of DOPC onto Hg from the G∣S interface and from a liposomal suspension. *J. Electroanal. Chem.* **516,** 73-82 (2001).

55. Green, N. G. & Jones, T. B. Numerical determination of the effective moments of non-spherical particles. *J. Phys. D: Appl. Phys.* **40,** 78-85 (2006).

56. Saville, D. A., Bellini, T., Degiorgio, V. & Mantegazza, F. An extended Maxwell–Wagner theory for the electric birefringence of charged colloids. *J. Chem. Phys.* **113,** 6974-6983 (2000).

57. Clarke, R. W., Piper, J. D., Ying, L. & Klenerman, D. Surface conductivity of biological macromolecules measured by nanopipette dielectrophoresis. *Phys. Rev. Lett.* **98,** 198102 (2007).

58. Al-Ahdal, S. A. *et al.* Dielectrophoresis of amyloid-beta proteins as a microfluidic template for alzheimer's research. *Int. J. Mol. Sci.* **20,** 3595 (2019).

59. Ing, N. L., El-Naggar, M. Y. & Hochbaum, A. I. Going the distance: Long-range conductivity in protein and peptide bioelectronic materials. *J. Phys. Chem. B* **122,** 10403-10423 (2018).
