# Supplementary material for: Precise capture and dynamic relocation of nanoparticulate biomolecules through dielectrophoretic enhancement by vertical nanogap architectures
Source: Nat Commun. 2020 Jun 4;11:2804. doi: 10.1038/s41467-020-16630-w (PMC7272609; doi:10.1038/s41467-020-16630-w)
Supplement: Supplementary file 3 — Description of Additional Supplementary Information [file 41467_2020_16630_MOESM3_ESM.pdf]

Description of Additional Supplementary Files

File Name: Supplementary Movie 1
Description: Frequency-dependent manipulation of 1-μm-diameter PS particles on VNE at AC voltages of f = 1 kHz, 10 kHz, 100 kHz, 1 MHz, and 10 MHz (Vpp = 2.5 V, 2x speed).

File Name: Supplementary Movie 2
Description: Trapping and releasing of 50-nm-diameter SUVs on VNE with the predefined pattern array of the KIST emblem under switching on (AC voltage of Vpp = 2 V and f = 100 kHz) and off.
